# Impact of Changing Socioeconomic Conditions on Family Caregiving Norms: Evidence from Japan

**DOI:** 10.3390/bs12120471

**Published:** 2022-11-23

**Authors:** Sayaka Fukuda, Sumeet Lal, Takuya Katauke, Mostafa Saidur Rahim Khan, Yoshihiko Kadoya

**Affiliations:** School of Economics, Hiroshima University, 1-2-1 Kagamiyama, Higashihiroshima 7398525, Japan

**Keywords:** Japanese eldercare, family caregiving norms, socioeconomic conditions, aging population

## Abstract

Eldercare is a major public health concern in many East Asian countries, including Japan, because of the ever-growing elderly population, and significant changes in family caregiving norms. The changes are due to global diffusion and the influence of socioeconomic and demographic shifts. Consequently, perceptions of the norm of family caregiving need investigation. We examined how demographic and socioeconomic factors influence the perception of family caregiving norms in Japan, using data from Osaka University’s preference parameter study. According to the results of the probit regression, age, education, full-time employment, marital status, the number of sons and daughters, interactions between females and age and females and full-time employment, and parents’ education are negatively related to the participants’ perceptions of family caregiving norms. Our results suggest that people traditionally perceived as caregivers are less likely to have a positive attitude towards family caregiving, despite the government’s efforts through Universal Long-Term Care Insurance, implemented in 2000. Therefore, authorities must reassess the role of families, explore alternative forms of community-based care, and provide more assistance to caregivers.

## 1. Introduction

Eldercare has become a major public health concern in Japan in recent decades, due to the unprecedented growth of the elderly population, which is expected to continue [1].

Furthermore, the number of older people who require activities of daily living and instrumental activities of daily living is steadily increasing [2]. For example, recent statistics from the Ministry of Health, Labour, and Welfare [3] show that the number of people who are officially recognized as a long-term care or support receiver has doubled to 6.223 million in 2016 from 2000. Although families provide the majority of eldercare worldwide, children’s perceived family caregiving norms toward their elderly parents vary across societies and countries [4]. Physical and psychological burdens and stress associated with caregiving activities have also affected caregivers’ attitudes toward caregiving norms and responsibilities [5,6,7,8]. Japan is a homogenous and traditional country with a deeply ingrained sense of elderly caregiving norms that are strongly rooted in intergenerational co-residence [9]. The influence of filial piety compels children of the elderly to perform even the most difficult caregiving tasks rather than seek assistance from institutional care [10]. However, despite the significant influence of traditional caregiving values, there has been a persistent change in how the filial caregiving norm operates [11]. The increasing number of elderly people in Japan over the years, combined with LTC reforms, massive demographic and socioeconomic changes, and the influence of the global diffusion process, has not only increased the demand for society to play a larger role in eldercare, but also influenced individuals’ perspectives on filial obligation norms [12]. As a result, it is critical to investigate various potential factors and how they relate to Japanese perceptions of the family caregiving norm. We hypothesize that in a homogeneous country such as Japan, where norms strongly shape people’s attitudes, there can be a major normative change in the perception of family caregiving of people with different demographic and socio-economic characteristics. While the implementation of the Long-Term Care Insurance (LTCI) system served as a partial buffer against the burden and stress, the strong influence of demographic changes, socioeconomic development, women’s participation in the formal labor market, and the influence of the global diffusion process could have affected the attitude of conventional caregivers toward eldercare.

Whether demographic changes, socio-economic development, or LTC reforms in Japan have influenced traditional caregiving values is an important but unresolved issue [13]. However, several emerging issues appear to challenge traditional caregiving norms and present a dilemma for choosing caregiving support. For example, in recent decades, the traditional living arrangement in which older people live with other family members in multigenerational households has been impulsively declining. According to the Cabinet Office of Japan [14], such living arrangements have decreased from 50.1% in 1980 to 12.2% in 2015. Given that the structural manifestation of traditional family norms prioritizes family over individual preferences [15], the declining trend in co-residence is an indication of a weakening norm in contemporary Japan. This is exacerbated by the clash of traditionalism and modernism, as Japan is at the forefront of social and economic change, exemplifying the transformation of cultural beliefs toward autonomy. For example, in Japan, it is believed that parental caregiving is primarily the responsibility of women, and the cultural norm requires the wife of the first son to fulfill all caregiving requirements for her in-laws [11,16]. However, in the 21st century, these traditional expectations have clashed with an increasing number of women working full-time away from home [17]. Thus, young women with more education and skills tend to negotiate living arrangements in pursuit of their own interests, and this movement eventually weakens co-residence with children. Furthermore, the moral tradition of parental caregiving was financially reinforced through the right of the firstborn child to inherit the entire wealth of the parents after the death of the father. However, this law was repealed after the Second World War, which had a profound negative impact on the perception of traditional caregiving norms [18]. Horioka [19,20] discovered that bequests for altruistic reasons are declining in Japan, and they are increasingly used to secure care and living expenses in old age. Other factors, such as the decline in birth rate, changes in family trends, including a greater tendency for people to marry later, and the increase in divorce rates, all have a significant impact on perceptions of family caregiving [21].

Several studies have examined people’s perception of eldercare in Japan. Khan, Watanapongvanich, and Kadoya [22], for example, examined people’s attitudes toward family caregiving in Japan and found that women, married individuals, and people earning a relatively low income are more likely to have a negative perception of eldercare. Furthermore, Ogawa and Retherford [23] used various predictor variables to examine women’s perceptions of parental caregiving norms. The findings revealed that younger women were less likely than older women to value filial caregiving. Moreover, although statistically insignificant, those with a high school diploma or higher view parental caregiving more favorably. Women who lived in urban areas and had husbands in professional fields such as medicine and education favored filial caregiving less than those who lived in rural areas or had husbands in non-professional fields such as electrical, plumbing, farming, and so on. According to Niimi [24], unmarried caregivers were significantly less happy than their married counterparts when providing eldercare to their parents. Tsutsui, Muramatsu, and Higashino [25] discovered that daughters-in-law and biological daughters had negative perceptions of eldercare norms, whereas the sons’ perceptions remained the most positive among family caregivers. This was due to both explicit (bequests) and implicit (preference for the son over the daughter) factors that influenced the perception of the sons as the primary caregivers. Furthermore, care recipients’ wives had the most negative perception of caregiving, which reflects the declining roles of daughters-in-law and the increasing roles of wives in Japan [22].

The decade-long massive socioeconomic development, combined with a rapidly aging population, has placed Japan in a critical stage where social norms for eldercare have been severely impacted, potentially having severe repercussions for old age support and putting additional financial pressure on the government’s healthcare budget. Identifying the factors that influence the perception of family caregiving norms becomes critical in this scenario so that policymakers can devise solutions to meet eldercare needs. Therefore, in this study, we examine the relationship between various socioeconomic and demographic factors and Japanese perceptions of family caregiving norms.

This study contributes to the existing literature in at least three ways. First, our findings demonstrate how demographic and socioeconomic conditions influence the changing perceptions of family caregiving norms in Japan. Second, this study includes some important social variables that have not been previously studied, such as religion, government responsibility, and social security, which could provide new dimensions to our understanding of family caregiving. Third, subsample analysis of various socioeconomic and demographic factors by age and sex could assist researchers in determining which groups of people are most receptive to changing family caregiving norms.

## 2. Data and Methods

### 2.1. Data

Our study uses data from the 2011 and 2013 waves of the Institute of Social and Economic Research at Osaka University’s preference parameters study (PPS), a 10-year panel survey that collected prospective information on individuals’ socioeconomic and demographic characteristics and preferences. Although we primarily used the 2013 dataset, some critical variables, such as respondents’ years of education and number of siblings, were readily available only in the 2011 wave. Therefore, we merged the two datasets to obtain these demographic variables. We excluded 1803 respondents who had missing data on some socioeconomic and demographic variables, bringing the final sample size in this study to 2534 or approximately 58% of valid respondents in the 2013 dataset (N = 4337).

### 2.2. Variables

The dependent variable in this study is “perception of family caregiving norm”. We have used the statement “children should take care of their parents” as a proxy to capture respondents’ attitude toward family caregiving norms in Japan. Respondents could answer this question on a scale of 1 to 5, with 1 being “completely agree” and 5 being “completely disagree”. Those who selected “completely agree” or “agree” (1 or 2 on the scale, respectively) were considered to have a positive perception of the norms, and those who answered “neither agree nor disagree”, “disagree”, or “completely disagree” (3, 4, or 5 on the scale, respectively) were considered to have a non-positive perception toward parental caregiving norms. “Perception of family caregiving norm” was coded as 1 for those who had a positive perception of parental caregiving norms and were more likely to rely on formal care in old age, and 0 for those who did not. Given that Japan is a highly collectivist country where individuals’ daily actions are guided by societal norms, we hypothesize that adherence to norms will play a positive role in meeting societal expectations for parental caregiving in light of the rapidly aging population.

The explanatory variables in this study have been divided into five groups. The first group comprises individual demographic characteristics, such as sex, age, respondent’s education level, religious level, employment status, marital status, as well as some important interaction terms such as sex with age and sex with full-time employment. The second group comprises potential caregiver variables, such as respondents’ number of siblings, number of sons, and number of daughters. The third group includes financial variables, such as the logs of household annual income and household financial assets. The fourth group comprises variables related to financial planning for old age, such as the proportion of living expenses that respondents will be able to cover with social security income after retirement, the perception of government’s role in taking care of financially dependent people, having savings for post-retirement living expenses, and having savings for long-term care. The last group of variables includes parent-related variables such as whether the respondents received an inheritance or monetary transfers in excess of 5 million JPY from their parents, parents’ education level, whether one parent requires care, and whether both parents require care. Most of our explanatory variables are comparable to those of Khan, Watanapongvanich, and Kadoya [22]. Table 1 presents the definitions of all the variables.

### 2.3. Descriptive Statistics

According to the descriptive statistics presented in Table 2, 56.5% of respondents have a positive perception of family caregiving norms. This sample was fairly distributed by sex, with 49.5% of the respondents being female, and the mean respondent age being 53.6 years. On average, respondents have 13.4 years of education; approximately 41.3% are employed full-time, 84.1% are married, and on average have two siblings in addition to one son and one daughter. Furthermore, 7.2% of respondents have strong religious beliefs, 50% have some form of social security, and 28.3% believe that the government should take care of people who are financially unable to support themselves. In terms of savings, whereas 61.4% of respondents have saved for a comfortable post-retirement life, 38.8% have sufficient savings for long-term care. Moreover, 18.5% of respondents have inherited a fortune worth more than 5 million JPY from their parents. On average, respondents’ parents have 10.9 years of education. Finally, 15.9% of respondents revealed that one of their parents requires long-term care, and 1.6% responded that both of their parents require long-term care.

Overall, we observe that people with different characteristics have different perceptions of family caregiving norms, as presented in Table 3 and Table 4.

Table 3 illustrates how perceptions of family caregiving norms differ across four major age groups. In general, age plays a significant role in determining people’s behavior; however, our ANOVA test results indicate otherwise. We observe that age has no effect on people’s perceptions of family caregiving norms. This result indicates that age is not a strong predictor on its own and that it could be combined with some other demographic variable to demonstrate a combined effect.

Table 4 shows that, at the 1% significance level, the percentage of females in favor of parental caregiving is lower than that of males. Similarly, a significant difference in employment status can be observed, with full-time workers having a more positive attitude toward family caregiving norms than non-full-time workers at the 1% significance level. This result implies that these respondents have a better financial situation and are willing to conform to the norm, potentially through paid professional caregiving services. Furthermore, there is a significant difference between the perceptions of married and unmarried respondents regarding family caregiving norms. It is noteworthy that the percentage of unmarried respondents who support parental caregiving is higher than the percentage of married respondents. This implies that an individual’s commitments or priorities change after marriage, and do not always move in favor of family caregiving.

### 2.4. Methodology

Using Equation (1), we examined the relationship between respondents’ perception of family caregiving norms and various demographic and socioeconomic characteristics. Since our dependent variable is binary, we conduct a probit regression to estimate the equation.

(1) $$Y_{i}=f\left(X_{i},\;\varepsilon_{i}\right)$$

where $Y_{i}$ is ith respondent’s perception of family caregiving norms, X is a vector of respondents’ individual characteristics and their relevant interaction terms, and ε is the error term.

Since we have used multiple independent variables to explain their possible relationship with family caregiving behavior, our model may suffer from high intercorrelations. For example, an individual with more years of education may have higher household wealth or be employed full-time. Therefore, we conducted correlation and multicollinearity tests (the results are available upon request). Across all models, the correlation matrix reveals a weak relationship between the relative movements of two variables (substantially lower than 0.70). Furthermore, the variance inflation factor tests of the explanatory variables are all less than 3, indicating that multicollinearity is present in all models but is insignificant.

We developed five models for Equation (1), each with a distinct control variable, which are depicted in Models 1, 2, 3, 4, and 5:

(1) $Perception\;of\;family\;caregiving\;norms_{i}$ = $\beta_{0}$ + $\beta_{1}female_{i}$ +$\beta_{2}age_{i}$ + $\beta_{3}age\_sq_{i}$ + $\beta_{4}years\_of\_education_{i}$ + $\beta_{5}religious\_level_{i}$ + $\beta_{6}fulltime\_employment_{i}$ + $\beta_{7}married_{i}$ + $\beta_{8}cross\_fem\&age_{i}$ + $\beta_{9}cross\_fem\&fulltime_{i}$ + $\varepsilon_{i}$;

(2) $Perception\;offamily\;caregiving\;norms_{i}$ = $\beta_{0}$ + $\beta_{1}female_{i}$ + $\beta_{2}age_{i}$ + $\beta_{3}age\_sq_{i}$ + $\beta_{4}years\_of\_education_{i}$ + $\beta_{5}religious\_level_{i}$ + $\beta_{6}fulltime\_employment_{i}$ + $\beta_{7}married_{i}$ + $\beta_{8}cross\_fem\&age_{i}$ + $\beta_{9}cross\_fem\&fulltime_{i}+\beta_{10}nb\_siblings_{i}$ + $\beta_{11}nb\_sons_{i}$ + $\beta_{12}nb\_daughters_{i}$ + $\varepsilon_{i}$;

(3) $Perception\;of\;family\;caregiving\;norms_{i}$ = $\beta_{0}$ + $\beta_{1}female_{i}$ + $\beta_{2}age_{i}$ + $\beta_{3}age\_sq_{i}$ + $\beta_{4}years\_of\_education_{i}$ + $\beta_{5}religious\_level_{i}$ + $\beta_{6}fulltime\_employment_{i}$ + $\beta_{7}married_{i}$ + $\beta_{8}cross\_fem\&age_{i}$ + $\beta_{9}cross\_fem\&fulltime_{i}+\beta_{10}nb\_siblings_{i}$ + $\beta_{11}nb\_sons_{i}$ + $\beta_{12}nb\_daughters_{i}$ + $\beta_{13}\log of\;household\;income_{i}$ + $\beta_{14}\log of\;household\;asset_{i}$ + $\varepsilon_{i}$;

(4) $Perception\;of\;family\;caregiving\;norms_{i}$ = $\beta_{0}$ + $\beta_{1}female_{i}$ + $\beta_{2}age_{i}$ + $\beta_{3}age\_sq_{i}$ + $\beta_{4}years\_of\_education_{i}$ + $\beta_{5}religious\_level_{i}$ + $\beta_{6}fulltime\_employment_{i}$ + $\beta_{7}married_{i}$ + $\beta_{8}cross\_fem\&age_{i}$ + $\beta_{9}cross\_fem\&fulltime_{i}+\beta_{10}nb\_siblings_{i}$ + $\beta_{11}nb\_sons_{i}$ + $\beta_{12}nb\_daughters_{i}$ + $\beta_{13}\log of\;household\;income_{i}$ + $\beta_{14}\log of\;household\;asset_{i}$ + $\beta_{15}social\_security_{i}$ + $\beta_{16}gvt\_responsibility_{i}$ + $\beta_{17}save\_retire_{i}$ + $\beta_{18}save\_ltc_{i}$ + $\varepsilon_{i}$;

(5) $Perception\;of\;family\;caregiving\;norms_{i}$ = $\beta_{0}$ + $\beta_{1}female_{i}$ + $\beta_{2}age_{i}$ + $\beta_{3}age\_sq_{i}$ + $\beta_{4}years\_of\_education_{i}$ + $\beta_{5}religious\_level_{i}$ + $\beta_{6}fulltime\_employment_{i}$ + $\beta_{7}married_{i}$ + $\beta_{8}cross\_fem\&age_{i}$ + $\beta_{9}cross\_fem\&fulltime_{i}+\beta_{10}nb\_siblings_{i}$ + $\beta_{11}nb\_sons_{i}$ + $\beta_{12}nb\_daughters_{i}$ + $\beta_{13}\log of\;household\;income_{i}$ + $\beta_{14}\log of\;household\;asset_{i}$ + $\beta_{15}social\_security_{i}$ + $\beta_{16}gvt\_responsibility_{i}$ + $\beta_{17}save\_retire_{i}$ + $\beta_{18}save\_ltc_{i}$ + $\beta_{19}parents\_inheritance_{i}$ + $\beta_{20}parents\_educ_{i}$ + $\beta_{21}one\_parentcare_{i}$ + $\beta_{22}both\_parentcare_{i}$ + $\varepsilon_{i}$.

## 3. Results

Table 5 presents the full-sample regression results of family caregiving norm perceptions. Starting with Model 1, certain distinct control variables are added to the subsequent model while retaining all other variables used in the previous model. This is carried out to ensure the robustness of the results and to assess the impact of the new control variables on existing ones. It is noteworthy that all variables are consistent in terms of signs and significance level across all models. According to our findings, females have a strong positive correlation with family caregiving behavior, indicating an increase in female commitments and roles to arduous parental caregiving in Japan, whether voluntary or involuntary. Religious beliefs, which influence people’s attitudes and perspectives, have a positive correlation with family caregiving behavior. Furthermore, full-time employment, log of household income, and government responsibility are all positively related to family caregiving behavior. By contrast, a negative relationship is observed between eldercare norms and being married, interaction terms of female and age, as well as female and full-time employment, and the number of sons. This implies a shift in the paradigm of parental expectations that sons should take care of them during old age, as sons’ attitudes toward eldercare are also heavily influenced by their marital and employment status.

Given that the categorization of various socioeconomic and demographic characteristics by age or sex could reveal significant findings on family caregiving perceptions, we conduct a subsample analysis of males and females stratified by age groups, in addition to using other socioeconomic and demographic factors to explain the phenomenon (Table 6). Important variables that were not previously significant in the full-sample analysis become significant in the subsample analysis. Specifically, we observe that age is negatively correlated with family caregiving behavior among females aged 51 and above, whereas age squared has a positive association within the same age group and sex. Years of education is negatively correlated with family caregiving norms only for females aged less than 40 and for those between 51 and 60. Interestingly, the number of daughters is negatively correlated with family caregiving behavior among females aged 60 and above. This implies that older females understand the tedious task of caring for elderly parents and would not want to burden their daughters with it in the future. Savings for long-term care and inheritance from parents are observed to be positively related to family caregiving perceptions, with the former being relevant for the oldest females and the latter being relevant for the youngest males. Furthermore, males between the ages of 51 and 60, whose parents had a higher level of education, are less likely to have a positive perception of family caregiving. All other variables that were significant in the full-sample analysis are also significant in the subsample analysis, with the exception that the significance level and signs of these variables vary by age and sex subordinates.

## 4. Discussion

Our findings reveal that various socioeconomic and demographic factors have had a significant impact on females’ perception of family caregiving norms. Under demographic characteristics, whereas age is negatively correlated, and age squared is positively correlated. This indicates that there is a non-linear negative relationship between age and perception of family caregiving norms, but only for females over the age of 50. As shown by our full-sample analysis, this association is also consistent with the interactions of factors, such as being female and age. However, this situation marks the beginning of the increasing influence of aging on female perceptions of caregiving norms as they age. Wives, adult daughters, and daughters-in-law most commonly care for the elderly. They are typically middle-aged, with a considerable proportion of them being over the age of 65 [26]. Given that the physical and mental health of older female caregivers can be affected, the level of support they receive to alleviate the burden of care can influence their perception of caregiving norms. According to Haya et al. [27], Japanese rural and suburban areas have limited resources, potentially affecting people’s caregiving burden/perception and quality of life. Furthermore, given the increased likelihood of females working outside the home as a result of a weakening patriarchal society [28], these older women who have been taught the value of personal freedom and human rights may come to perceive caring for the elderly/partner as overly burdensome [29]. Alternatively, at some point in time, the aging process may begin to worsen these women’s health problems, which may not only change their perception, but also signal the need to take care of them in the short or medium term [30].

Furthermore, individuals with more education are less likely to have a positive attitude toward family caregiving norms. Specifically, females under the age of 40 and those between the ages of 51 and 60 have this perception. These findings are supported by the fact that most educated Japanese women are able to uncover hidden assumptions about eldercare when they are young, before marriage, and also during married life, typically when they have reached mid-life and their husband’s parents have grown old [9]. As a result of female empowerment through education and the country’s growing era of autonomy, highly educated females are financially independent, and capable of pursuing their interests for subjective welfare by negotiating living arrangements with their husbands rather than considering traditional caregiving norms as their obligations [31]. In the full-sample analysis, we observed that people in full-time employment are more likely to have a positive perception of family caregiving norms. However, in the subsample analysis, we observed that full-time employment has a positive impact on the perceptions of family caregiving norms among males aged 60 and over, but a negative impact on females aged 41 to 50 years and 60 years and over. As shown in the full-sample analysis, this association is consistent with the interaction term of female and full-time employment. This implies that as more women enter the workforce as full-time employees, they will be more hesitant to provide filial care. According to Lee [17], Japanese women negotiate not only within-cohort conflicts regarding norms and cultural beliefs, but also inter-cohort conflicts regarding norms and beliefs correlated with the household governance system, in order to accommodate their full-time paid employment. This result is also consistent with the findings of Saito [21] and Long and Harris [32]. Researchers such as Kikuzawa and Uemura [29] have demonstrated the unfavorable impact of caregiving norms on women’s employment by discovering that women in regular employment who provided five or more hours of care and those who had provided any number of hours of care in the previous year were more likely to stop working or change jobs than their non-caregiving counterparts.

Furthermore, consistent with previous findings, individuals with greater religious beliefs are more likely to favor family caregiving norms. According to Verbakel [33], religious individuals are more likely to be informal caregivers than non-religious individuals. We discover that religious level is positively associated with favorable perceptions of family caregiving norms among females over 60. In general, more females than males are religious and spiritual in Japan, and this religious personality shapes a favorable perception toward filial caregiving [34]. Moreover, these females were most probably caregivers when they were younger, and as a result, they will have the natural instinct that their children should do the same for them when they grow old. Overall, the difference in perceptions may be influenced by a generational gap, as evidenced by Hwang, Yoon, Brown, and Silverstein [35], who revealed that people born in the early 1970s and 1980s are more religious and likely to strongly follow filial norms than people born in the 21st century.

Married people are less likely to have a positive perception of traditional caregiving norms. Due to Japan’s long-term employment and seniority system, males between the ages of 41 and 50 tend to hold higher positions in their organization. These men, due to extreme workload, tend to neglect filial caregiving [36]. Furthermore, we observe that females over the age of 60 are less likely to have a positive perception of family caregiving norms. According to Japanese traditions, the wife of the first son is expected to fulfill all hands-on caregiving responsibilities for her in-laws [11], and this arrangement worked fairly well in the past. These responsibilities have burdened women after marriage, both physically and mentally [37], contributing to their negative attitude toward family caregiving [22]. Consequently, women who have had to bear the burden of family caregiving will not be willing to burden their children with the same responsibilities.

Potential caregiving variables, such as the number of sons and daughters, are less likely to elicit a positive perception toward family caregiving norms. This is true for females over the age of 60 in terms of number of daughters. The deterioration in subjective well-being and health of aging married women as a result of extreme caregiving responsibilities contributes significantly to the building a non-positive attitude towards family caregiving norms [38], as well as towards the prospect of having their daughters care for them as they grow older. The number of sons also has the same association, but only among females aged 41 to 50. This finding is consistent with that of Khan, Watanapongvanich, and Kadoya [22] that the more sons a couple had, the more likely they were to have a negative attitude toward receiving eldercare from them. According to Horioka [39], changing circumstances have caused Japanese people to rely less on their sons for family care, and more on formal care. Although inconsistent with previous research [21,25], our study reveals that societal expectations do not remain static over the course of a person’s life but rather evolve in response to changing social context.

In terms of financial variables, we observe individuals with higher household income to have a more positive perception of family caregiving norms. This association is stronger in the female subsample aged 51 to 60. We consider financially well-off people to have better mental health and more readily available resources to access and pay for professional or formal care in their household, rather than providing informal care themselves, which can be stressful [40]. Therefore, it is not unusual to discover this positive perception among female respondents in their 50s who typically provide family care. The only difference is that while they do not actively provide hands-on care, they may do so indirectly by employing others to do it [41].

Financial planning variables, such as government responsibility, elicit positive perceptions of family caregiving norms. Specifically, men aged 51 and above, as well as women between the ages of 41 and 50 are significantly influenced by government intervention, which would not only increase their capacity to provide effective informal caregiving services, but also reduce their burden and stress [42]. It is noteworthy that these people, who may be working in full-time jobs as well as taking responsibility for filial caregiving, are also ageing, which reduces their physical ability to provide effective eldercare. However, government intervention in financial, physical, or any other forms may help to incentivize and improve the perception regarding caregiving. Several studies have demonstrated that older people place more responsibility on the government to provide assistance or care to those in need [43], and this formal caregiving complements informal caregiving to a certain extent, eventually putting caregivers in a better position and positively influencing their perceptions [44]. We discover that savings for long-term care have a positive effect on older female respondents’ perceptions of family caregiving norms. We believe that those who save for long-term care prefer informal caregiving over formal care services. Only older women over the age of 60 had this idea in our study, indicating that traditional family caregiving norm is weakening among other age groups and sex who plan to save or may have saved enough for long-term formal caregiving services [45]. Furthermore, according to Niimi and Horioka [46], parental care responsibilities tend to emerge relatively late in life, often after retirement, and that the financial burden of parental care may become a relevant issue when analyzing the elderly’s wealth decumulation behavior. Thus, we believe that older women who save for long-term care may use the money as an incentive to motivate their children to care for them as well as support themselves in the future.

Variables related to parents, such as parental inheritance, influence the perception of family caregiving norms. This association is particularly strong among men who are less than 40 years old. According to Horioka et al. [47], Japanese men are more likely to live with (or near) their elderly parents and/or provide care and attention to them if they expect a bequest from them. This observation is supported by our study, in which we assume that younger men may value their heritage more since they have fewer life experiences and income, influencing them to have a positive perception of filial caregiving [48]. We also discover that parents’ higher years of education tend to discourage positive perceptions of family caregiving norms among middle-aged males. According to He and Chou [49], at the age of 50 or 60, men who also experience diminishing health due to aging or who are heavily burdened with employment or other social services may view higher parental education as a mechanism through which parents can independently begin to prepare or have already planned for long-term care. This is due to the fact that a higher education would have guaranteed them a good lifelong income through continued employment. As a result, an individual believes that their parents do not require any assistance or support, either physically or financially, as they are qualified or experienced enough to arrange for formal care on their own, or are well aware of the risks if they have not arranged for any formal care [50].

In general, our findings suggest that demographic changes coupled with socioeconomic development and the global social diffusion process influenced the traditional caregiving norms maintained by family caregivers. If primary family caregivers continue to have a negative attitude towards providing eldercare, there would be a serious consequence on the long-term care market. As a buffer against the pressure on family caregiving stress and burden, Japan introduced the LTCI system in 2000. However, our results imply that government support including LTCI was not sufficient to address the ever-growing family caregiving burden resulting in eroding the virtue of family care to some extent. For example, Arai and Kumamoto [51] found that family caregiving burden did not decrease despite the LTCI has increased the use of LTC. Moreover, Saito [21] found that caregiving roles and responsibilities assumed by families and supports provided by the society is still unbalanced and has not removed the families’ caregiving burden even after the introduction of the LTCI system. Thus, the evolving family caregiving attitude should be addressed properly so that government effort towards the LTC could be sufficiently complemented by families.

There are several limitations to this study that should be considered when interpreting the findings. First, this study examined the association between the perception of family caregiving norms and several demographic and socioeconomic characteristics using data from 2011 and 2013. Since this is not the most recent dataset, the socioeconomic and demographic changes that have occurred in Japan in recent years may impact our results. Second, our study does not include certain critical variables, such as daughters-in-law, sons’ marital status, or location, as questions involving these variables were not included in the questionnaire. These variables would have provided more dimensions and insight into how changing family structure or setup influences an individual’s attitude toward family caregiving norms. Third, since this was a cross-sectional study, we were unable to make any causal inferences. A longitudinal study, therefore, is needed to capture how perceptions of caregiving norms change over time as a result of changing social strata and the influence of the global diffusion process.

## 5. Conclusions

This study investigated how changing socioeconomic conditions influence the family caregiving norms in Japan by dividing the population into different age groups based on sex, and including important socioeconomic and demographic variables to elucidate the phenomenon. We assumed that in a homogeneous country such as Japan where the norm strongly shapes the attitude of the people, there can be a major normative change in the perception of family caregiving due to the influence of socioeconomic and demographic changes, which impacts different groups of people in a unique manner. Most of our results are in line with expectations and we find that regression coefficients such as age, education years, full-time employment, being married, number of sons and daughters, both interaction terms and parent’s education are negatively related to perception of the norm of family caregiving; however, the significance of these results vary between sexes and various age groups. Moreover, although not consistent across subsamples, we find that age squared, religious level, log of household income, government responsibility, savings for long-term care and inheritance from parents have positive correlation with family caregiving norms. Although previous studies show that those who are females, married and are one of the many sons and daughters are traditionally perceived to be potential caregivers [52,53], our results show otherwise, whereby it is mostly this group of people that exhibit a negative tendency toward family caregiving norms.

The findings of this study have significant implications for policymakers, government officials, and researchers. In light of rapid socioeconomic development, demographic changes, and the influence of the global diffusion process, the current state of family caregiving in Japan requires special attention, as these changes have a significant impact on the perception of family caregiving norms. Since many Japanese people now prefer autonomy over collectivism, expecting family members to follow traditional norms and continue taking care of the elderly would be a mistake, given that the caregiving norm now prevents them from pursuing careers or other self-interests that maximize their welfare. Thus, we suggest for more support for family caregivers in addition to the existing LTCI’s efforts to reduce caregiving burden. The government still needs to respond to the transformation of the people’s perception of family caregiving norms. Reassessing the role of families, exploring alternative forms of community care, and providing assistance to caregivers in light of deteriorating social norms is a critical step toward mitigating long-term care costs and filling the ever-increasing gap between demand for and supply of caregiving resources.

## Figures and Tables

**Table 1:** Variable definitions.

Variables	Definition
Perception of family caregiving norms	Dependent variable Binary variable which equals 1 when the respondent completely agrees or agrees with the statement “Child(ren) should take care of their parents when they require long-term care”, and 0 otherwise.
	Independent variables
female	equals 1 if the respondent is a female, and 0 is the
age	age of the respondent
age_sq	age squared
religious_level	equals 1 if respondent holds strong religious beliefs
years_of_education *	number of years of education
fulltime_employment	equals 1 if the respondent has full-time employment
married	equals 1 if the respondent is married
cross_fem&age	interaction term between sex and age
cross_fem&full-time	interaction term between sex and full-time employment
nb_siblings *	number of siblings
nb_sons	number of sons
nb_daughters	number of daughters
log of household income	natural log of annual household income
log of household asset	natural log of household financial assets
social_security	Proportion of living expenses that the respondent will be able to cover using social security income after retirement
gvt_responsible	equals 1 if the respondent either agrees or strongly agrees with the statement “it is the government’s responsibility to take care of themselves financially”
save_retire	equals 1 if the respondent has savings to meet post-retirement living expenses
save_ltc	equals 1 if the respondent has savings for long-term care
parents_inheritance	equals 1 if the respondent has received an inheritance or monetary transfer of 5 million JPY or more from parents
parents_educ	average years of education of parents
one_parentcare	equals 1 if one parent requires care
both_parentcare	equals 1 if both parents require care

Note: The symbol * indicates data from the 2011 dataset.

**Table 2:** Descriptive statistics.

Variable	Mean (Percentage for Binary Variables)	Std. Dev.	Min	Max
Perception of family caregiving norm	56.51%	-	0	1
female	49.45%	-	0	1
age	53.6287	12.4831	24	80
age_sq	3031.7980	1323.4910	576	6400
year_of_education	13.4100	2.1355	9	21
religious_level	7.22%	-	0	1
full-time_emplyment	41.36%	-	0	1
married	84.14%	-	0	1
cross_fem&age	26.0868	27.7711	0	80
cross_fem&fulltime	10.66%	-	0	1
nb_siblings	1.8587	1.2528	0	9
nb_sons	0.9988	0.8740	0	5
nb_daughters	0.8887	0.8285	0	5
household income	6,339,187	3,803,178	500,000	21,000,000
log of household income	15.4769	0.6501	13.1224	16.8600
household asset	14,500,000	19,800,000	1,250,000	125,000,000
log of household asset	15.7675	1.2344	14.0387	18.6438
social_security	0.5084	0.2728	0.05	1
gvt_responsible	28.30%	-	0	1
save_retire	61.48%	-	0	1
save_ltc	38.83%	-	0	1
parents_inheritance	18.59%	-	0	1
parents_educ	10.9786	1.9581	9	18.5
one_parentcare	15.98%	-	0	1
both_parentcare	1.62%	-	0	1
Total observations		2534		

**Table 3:** Perceptions of family caregiving norms by age group.

Perceptions of Family Caregiving Norm	Age (in Years)				Total
	Less Than or Equal to 40	41–50	51–60	Greater Than 60	
0	165	264	290	383	1102
	41.04%	41.06%	45.17%	45.22%	43.49%
1	237	379	352	464	1432
	58.96%	58.94%	54.83%	54.78%	56.51%
Total	402	643	642	847	2534
	100%	100%	100%	100%	100%
F-statistics	F = 1.43				

**Table 4:** Perceptions of family caregiving norms by demographic characteristics.

Perception of Family Caregiving Norm	Sex		Full-Time Employment		Married		Total
	Female	Male	Yes	No	Yes	No	
0	630	472	410	692	953	149	1102
	50.28%	36.85%	39.12%	46.57%	44.70%	44.70%	43.49%
1	623	809	638	794	1179	253	1432
	49.72%	63.15%	60.88%	53.43%	55.30%	62.94%	56.51%
Total	1253	1281	1048	1486	2132	402	2534
	100%	100%	100%	100%	100%	100%	100%
Mean Difference	t = −6.8805 ***		t = 3.7322 ***		t = −2.8358 ***		

Note: The symbol *** indicates *p* < 0.01.

**Table 5:** Probit regression results.

Variables	Dependent Variable: Perception of Family Caregiving Norms				
	Model 1	Model 2	Model 3	Model 4	Model 5
female	0.5864 **	0.6260 **	0.6103 **	0.6088 **	0.6122 **
	−0.2719	−0.273	−0.2739	−0.2751	−0.2753
age	0.0119	0.0131	0.0109	0.0088	0.0076
	−0.0167	−0.017	−0.0171	−0.0172	−0.0179
age_sq	−0.0001	−0.0001	−0.0001	0	0
	−0.0002	−0.0002	−0.0002	−0.0002	−0.0002
year_of_education	0.0006	−0.0007	−0.0093	−0.0121	−0.0145
	−0.0126	−0.0128	−0.0134	−0.0136	−0.0146
religious_level	0.2203 **	0.2269 **	0.2413 **	0.2129 **	0.2181 **
	−0.0998	−0.1002	−0.1006	−0.1017	−0.1017
full-time_employment	0.1833 **	0.1861 **	0.1555 *	0.1642 *	0.1655 *
	−0.0857	−0.0859	−0.0873	−0.0876	−0.0878
married	−0.2463 ***	−0.2003 **	−0.2304 ***	−0.2045 **	−0.2043 **
	−0.0762	−0.0837	−0.085	−0.0855	−0.0856
cross_fem&age	−0.0150 ***	−0.0156 ***	−0.0156 ***	−0.0155 ***	−0.0157 ***
	−0.0045	−0.0045	−0.0045	−0.0045	−0.0046
cross_fem&fulltime	−0.3202 **	−0.3250 ***	−0.3307 ***	−0.3469 ***	−0.3468 ***
	−0.1245	−0.1246	−0.1249	−0.1258	−0.1259
nb_siblings		−0.0147	−0.0134	−0.0131	−0.0124
		−0.0222	−0.0223	−0.0224	−0.0225
nb_sons		−0.0727 **	−0.0750 **	−0.0744 **	−0.0715 **
		−0.0329	−0.0333	−0.0334	−0.0334
nb_daughters		0.0029	0.0021	0.0041	0.0067
		−0.0343	−0.0346	−0.0348	−0.0348
log of household income			0.0880 *	0.0901 *	0.0891 *
			−0.046	−0.0462	−0.0463
log of household asset			0.0206	0.0175	0.0185
			−0.0239	−0.0262	−0.0265
social_security				−0.1695	−0.1707
				−0.1055	−0.1058
gvt_responsible				0.2569 ***	0.2556 ***
				−0.057	−0.0571
save_retire				−0.0091	−0.0077
				−0.0689	−0.069
save_LTC				0.0834	0.0855
				−0.0687	−0.0688
parents_inheritance					−0.027
					−0.0704
parents_educ					0.0074
					−0.0158
one_parentcare					0.0323
					−0.0742
both_parentcare					0.2973
					−0.213
Constant	−0.0101	−0.0045	−1.4613 *	−1.3904 *	−1.4280 *
	−0.4655	−0.4798	−0.767	−0.788	−0.811
Observations	2534	2534	2534	2534	2534
Log likelihood	−1696	−1693	−1690	−1677	−1676
Chi2 statistics	76.79	82.67	88.33	112.3	116
*p*-value	0	0	0	0	0

Note: Robust standard errors are presented in parentheses. The symbols ***, **, and * denote *p* < 0.01, *p* < 0.05, and *p* < 0.1, respectively.

**Table 6:** Perception of family caregiving norms by sex and age: subsample regression results.

Variables	Dependent Variable: Perception of Family Caregiving Norms							
	Male				Female			
	Age ≤ 40	Age 41–50	Age 51–60	Age > 60	Age ≤ 40	Age 41–50	Age 51–60	Age > 60
age	−0.1588	0.0535	−0.1376	0.2232	−0.2579	−0.1660	−2.2229 **	−0.8432 *
	(0.3184)	(0.9102)	(1.2288)	(0.3668)	(0.3198)	(0.9177)	(1.1011)	(0.4434)
age_sq	0.0022	−0.0008	0.0009	−0.0016	0.0038	0.0018	0.0201 **	0.0060 *
	(0.0048)	(0.0100)	(0.0111)	(0.0027)	(0.0048)	(0.0101)	(0.0099)	(0.0032)
year_of_education	0.0468	−0.0420	0.0266	0.0339	−0.1124 *	−0.0400	−0.1482 ***	−0.0101
	(0.0525)	(0.0396)	(0.0384)	(0.0286)	(0.0581)	(0.0541)	(0.0535)	(0.0476)
religious_level	0.7674	0.2597	0.1759	0.0168	0.4318	0.3077	0.3053	0.3431 *
	(0.5651)	(0.4935)	(0.2832)	(0.2416)	(0.4922)	(0.3218)	(0.2444)	(0.2084)
fulltime_employment	0.2645	−0.0509	0.1278	0.2985 **	0.1248	−0.3745 **	−0.0616	−0.5886 *
	(0.2701)	(0.2182)	(0.2154)	(0.1515)	(0.2206)	(0.1766)	(0.1687)	(0.3106)
married	−0.2186	−0.5329 *	−0.1447	0.3186	−0.2566	−0.3926	−0.1754	−0.5039 ***
	(0.3026)	(0.2736)	(0.2740)	(0.2805)	(0.3160)	(0.2701)	(0.2560)	(0.1929)
nb_siblings	−0.0747	−0.1290	−0.0934	0.0256	−0.0056	0.0203	−0.0532	−0.0267
	(0.1432)	(0.1030)	(0.0745)	(0.0406)	(0.1224)	(0.0735)	(0.0689)	(0.0505)
nb_sons	0.2664	0.0060	−0.1539	−0.0430	−0.0458	−0.1689 *	−0.1104	−0.1289
	(0.1634)	(0.1062)	(0.0947)	(0.0798)	(0.1322)	(0.0965)	(0.0912)	(0.0975)
nb_daughters	0.1365	0.1321	−0.0354	0.0343	0.0388	0.1190	−0.1333	−0.1766 *
	(0.1416)	(0.0960)	(0.0952)	(0.0828)	(0.1554)	(0.1100)	(0.0949)	(0.0979)
log of household income	−0.0885	0.0275	−0.0151	0.0205	0.2339	0.2057	0.3050 **	0.1520
	(0.2242)	(0.1341)	(0.1379)	(0.1109)	(0.2029)	(0.1525)	(0.1264)	(0.1229)
log of household asset	−0.0815	−0.0102	0.0733	−0.0095	−0.1009	0.0720	0.0582	0.0091
	(0.1058)	(0.0810)	(0.0804)	(0.0622)	(0.1030)	(0.0766)	(0.0776)	(0.0722)
social_security	−0.6571	−0.3628	−0.2734	−0.1656	0.4497	−0.2046	0.0505	−0.2228
	(0.4735)	(0.3709)	(0.3350)	(0.2437)	(0.4308)	(0.3489)	(0.2867)	(0.2421)
gvt_responsible	0.3889	0.1207	0.4628 ***	0.3876 ***	0.1965	0.3142 *	0.0895	0.1573
	(0.2466)	(0.1719)	(0.1714)	(0.1355)	(0.2174)	(0.1649)	(0.1619)	(0.1547)
save_retire	−0.0949	0.1490	0.0596	−0.3338	−0.2512	0.1884	0.2072	−0.0783
	(0.2686)	(0.1840)	(0.1899)	(0.2038)	(0.2241)	(0.1798)	(0.2004)	(0.2441)
save_LTC	0.0594	0.0915	0.3019	0.1334	0.0344	−0.2982	−0.1009	0.4616 **
	(0.3634)	(0.2149)	(0.1925)	(0.1628)	(0.3370)	(0.1992)	(0.1747)	(0.1900)
parents_inheritance	1.1766 *	−0.1001	0.0253	0.0846	0.0509	−0.1447	−0.0994	−0.1916
	(0.6246)	(0.2201)	(0.1835)	(0.1282)	(0.5663)	(0.3480)	(0.2008)	(0.1745)
parents_educ	−0.0079	0.0185	−0.0864 *	−0.0199	0.1019	0.0687	0.0457	0.0218
	(0.0627)	(0.0428)	(0.0442)	(0.0375)	(0.0627)	(0.0481)	(0.0444)	(0.0462)
one_parentcare	−0.3209	−0.2481	0.1721	−0.1131	–	−0.0172	−0.0513	0.0483
	(0.6354)	(0.2336)	(0.1910)	(0.1878)	–	(0.2318)	(0.1640)	(0.1906)
both_parentcare	–	1.0466	0.3181	0.9355	–	0.0848	0.1866	0.3041
	–	(0.6378)	(0.4749)	(0.5786)	–	(0.7132)	(0.4373)	(0.6305)
Constant	5.0613	0.3809	5.0729	−7.9860	2.6616	−0.2803	57.3959 *	27.3734 *
	(6.1465)	(20.7687)	(34.0652)	(12.6027)	(6.1004)	(20.8995)	(30.4352)	(15.3626)
Observations	181	317	311	472	211	326	331	375
Log likelihood	−112.9	−197.1	−186.3	−302.5	−136.4	−211.6	−216.7	−242.3
Chi2 statistics	19.96	16.31	30.72	21.65	16.13	25.17	22.01	28.43
*p*-value	0.335	0.637	0.0433	0.302	0.515	0.155	0.284	0.0754

Note: The robust standard errors are presented in parentheses. The symbols ***, **, and * denote *p* < 0.01, *p* < 0.05, and *p* < 0.1, respectively.

## Data Availability

Data are available upon request.
